# Exploring the feasibility of introducing triple artemisinin-based combination therapy in the malaria treatment policy in Vietnam

**DOI:** 10.1186/s12936-023-04763-4

**Published:** 2023-10-28

**Authors:** Van Anh Thi Cao, Thieu Quang Nguyen, Duong Le Quyen, Wouter P. C. Boon, Ellen H. M. Moors, Arjen M. Dondorp, Freek de Haan, Chanaki Amaratunga

**Affiliations:** 1 The University of North Carolina Project Office, Hanoi, Vietnam; 2 National Institute of Malariology, Parasitology and Entomology, Hanoi, Vietnam; 3 World Mosquito Program, Ho Chi Minh, Vietnam; 4 Copernicus Institute of Sustainable Development, Utrecht University, Utrecht, The Netherlands; 5 Mahidol Oxford Tropical Medicine Research Unit, Faculty of Tropical Medicine, Mahidol University, Bangkok, Thailand; 6 Centre for Tropical Medicine and Global Health, Nuffield Department of Medicine, University of Oxford, Oxford, UK

**Keywords:** Triple artemisinin-based combination therapy (TACT), Introduction strategies, Drug resistance

## Abstract

**Background:**

This study investigates the processes regarding changing malaria treatment policies in Vietnam. Moreover, it explores the feasibility of introducing triple artemisinin-based combination therapy (TACT) in Vietnam to support the national malaria control and elimination plan.

**Methods:**

Data were collected via 12 in-depth interviews with key stakeholders, combined with a review of policy documents.

**Results:**

TACT is considered as a useful backup strategy in case future treatment failures with current artemisinin-based combination therapy (ACT) would occur. Moreover, TACT is also considered as a promising strategy to prevent the re-establishment of malaria. However, regulatory procedures and implementation timelines for TACT were expected to be lengthy. Therefore, strategies to engage national decision-makers, regulators, and suppliers should be initiated soon, stipulating the benefits of TACT deployment. In Vietnam, a procedure to apply for an import permit without registration that has previously been applied to the introduction of artesunate-pyronaridine was proposed to accelerate the introduction of TACT. Global-level support through the World Health Organization recommendations and prequalification were considered critical for supporting the introduction of TACT in Vietnam.

**Conclusions:**

Appropriate approach strategies and early stakeholder engagement will be needed to accelerate the introduction of TACT in Vietnam.

**Supplementary Information:**

The online version contains supplementary material available at 10.1186/s12936-023-04763-4.

## Background

Over the last two decades, there has been a significant decrease in malaria morbidity and mortality in Vietnam. Of 63 provinces, 42 have eliminated malaria. Moreover, total malaria cases in the country reduced from 4,665 in 2019 to 455 in 2022. Of these, the number of cases identified as *Plasmodium falciparum* reduced from 3110 to 272, with *Plasmodium vivax* and mixed-species infections constituting the remainder [1]. In line with these developments, Vietnam has set targets of eliminating *P. falciparum* malaria by 2025 and all species of malaria by 2030 [2]. Nevertheless, 7 million people in Vietnam still reside in areas at risk of malaria.

Dihydroartemisinin-piperaquine (DHA-PPQ) has been deployed as the first-line therapy for the treatment of uncomplicated falciparum malaria in Vietnam since 2005 [3, 4]. However, delayed parasite clearance after treatment with DHA-PPQ was detected in Binh Phuoc province in 2009. Subsequent surveillance also revealed delayed parasite clearance in Gia Lai province (2010), Dak Nong province (2011), Quang Nam province (2012), Khanh Hoa province (2014) and Ninh Thuan province (2015) [5, 6]. In response to treatment failures with DHA-PPQ [7, 8], artesunate-pyronaridine (PA) was officially introduced in 2020 as an alternative treatment regimen in Vietnam for areas where treatment failure following DHA-PPQ exceeded 10% [9]. This was in line with the World Health Organization (WHO) recommendation stipulating that countries should switch first-line therapies once treatment failures exceed 10% [5, 6]. Although PA is an effective treatment for *P. falciparum*, a relatively high proportion of patients remained parasitemic on D3 (24.0%—44.9%) [10, 11] calling for continuous monitoring of PA efficacy in Vietnam. This is worrisome because there are very few treatment options remaining if PA fails. Moreover, there is an ongoing risk of artemisinin resistance emerging and/or spreading to other provinces and beyond, which could cause a resurgence of malaria, similar to the recent outbreak observed during 2020 – 2021 in Lao PDR [12]. Therefore, alternative treatment strategies are urgently required to prevent artemisinin and subsequent partner drug resistance from spreading further and thereby to enable Vietnam to achieve the national malaria elimination target.

One promising solution to address the threat of artemisinin and partner drug resistance is the introduction of triple artemisinin-based combination therapy (TACT) [13]. Some triple artemisinin-based combinations are being developed and tested as a strategy to improve treatment efficacy in areas of drug resistance and to prevent further emergence and spread of resistance. The rationale is that combining artemisinin with two carefully selected partner drugs will extend the therapeutic lifetime of each compound because the probability that the parasite can develop resistance to three drugs is lower than developing resistance to two drugs. However, the potential future roll-out of TACT is likely complicated [14], despite the supporting results of the current clinical trials and mathematical modelling studies [15–18].

Vietnam has been forced to switch first-line malaria therapies as a response to emerging drug resistance [4, 9]. The study aimed to understand the processes regarding changing malaria treatment policies in Vietnam, more specifically to explore the feasibility of introducing TACT in the country. Which can also benefit other countries that struggle with updating treatment practices in an era of declining malaria cases.

## Methods

### Study setting

The Ministry of Health (MoH) is responsible for the national malaria control and elimination program, including formulation of policies and strategies. The National Institute of Malariology, Parasitology and Entomology (NIMPE) in Hanoi is responsible for development, coordination and technical oversight of the national program in collaboration with two regional Institutes of Malariology, Parasitology and Entomology (IMPEs) in Quy Nhon and Ho Chi Minh. The provincial, district, and commune levels are responsible for implementation of activities following the guidance of the national program. Malaria case detection and treatment are provided free of charge at the commune level and in public healthcare facilities. Malaria cases detected by private health care providers are referred to the public sector for treatment. Malaria control activities are supported financially by the Global Fund to fight AIDS, Tuberculosis and Malaria (GFATM). Technical support is provided by the WHO. The Drug Administration of Vietnam (DAV) under the management of MoH is responsible for drug regulation in Vietnam.

In the most updated national malaria treatment guideline issued in 2020, DHA-PPQ was prescribed as first-line treatment for uncomplicated cases of falciparum malaria. For *P. falciparum* cases with failure following DHA-PPQ, PA or artesunate–mefloquine (ASMQ) are the alternative treatment. In areas with evidence of > 10% DHA-PPQ treatment failure, PA and ASMQ became the first choices [9].

### Research design and data collection

A qualitative method approach was employed to explore processes regarding changing malaria treatment policies in Vietnam. Data were collected through 12 in-depth interviews, conducted as part of the market positioning work package of the Development of Triple Artemisinin Combination Therapies (DeTACT) project in Vietnam [13, 19].

Additional supporting documents to verify data collected during interviews were sourced from online platforms of the MoH, NIMPE and IMPEs, the WHO and through other government portals. The interviews were conducted with 12 key stakeholders from August to November 2022. Respondents are affiliated to different organizations and institutes, but all are involved in implementing or coordinating malaria treatment policies in Vietnam, and were recruited as follows: 1 from MoH, 6 from NMP, 4 from non-government organizations (NGO), and 1 from an international pharmaceutical company. The semi-structured guidelines for interviews were designed to allow for thematic analysis on the malaria treatment policy change while maintaining flexibility for additional topics to be brought up by respondents (Additional file 1: Annex S1). Data collection was iterative; insights from previous interviews were included in subsequent interviews. Some interviews were organized in-person while the rest were conducted using Microsoft Teams. Each interview lasted approximately 60 min. All interviews were conducted in Vietnamese and transcribed verbatim for analysis. Written notes were made to capture major issues in addition to verbal utterances and nuances.

### Data analysis

The Vietnamese transcripts were translated to English by professional translators with experience in the medical domain. The translated transcripts were then uploaded to NVivo12 Pro software and subjected to a process of coding. The coding process comprised both deductive and inductive techniques. A codebook was constructed based on the prior thematic analysis (deductive). New themes were developed for data that did not match the existing codes but were considered relevant for the study (inductive). The coding was done first by one researcher (VA), then validated by another (QD). These two researchers are fluent in both English and Vietnamese, which enabled them to examine the Vietnamese transcripts when needed. In a second round of coding, all themes were merged into overarching categories and storylines were written.

## Results and discussion

### Requirements for clinical trials

The Vietnam national legislative documents indicate that a foreign drug for which qualified safety and efficacy assessments from clinical trials are available—regardless of where the clinical trials were conducted—and has been accepted for sale in at least one country can be considered for circulation in Vietnam [20, 21]. However, most interview respondents were not aware of these procedures and assumed that results from clinical trials in Vietnam are necessary for a drug to be granted a license in the country. The respondents were concerned that efficacy and safety data collected outside Vietnam would not convince relevant decision makers in the country. To support their claim, interviewees referred to the introduction of PA in Vietnam. Before PA was used in the NMP from 2019, it was tested in several provinces in the Central Highlands of Vietnam despite the published results of international clinical trials for PA since 2008 [22, 23].

Normally, the pioneers must evaluate the efficacy and safety of this drug in Vietnam. Because later, according to the current process, the drugs are said to be effective somewhere over, people do not care, when it is introduced in Vietnam, there must still be a trial and evaluation of safety, efficacy, and safety of that drug in Vietnamese patients … The clinical trial is a must. The good results from this will be the basis for the (national malaria) program to propose for implementation of the malaria treatment… (Representative of NMP)

So, to put it in Vietnam, we need to have a protocol, even though it has been assessed in many research places, but when it is introduced into Vietnam, it still needs a re-assessment. (Representative of NGO)

In Vietnam, clinical trial proposals must be reviewed by the MoH’s Scientific and Ethical Committee before commencement. Trials must be implemented in compliance with the approved proposal and Good Clinical Practices (GCP) recognized by the MoH, such as International Council for Harmonization of Technical Requirements for Pharmaceuticals for Human Use—Guideline for Good Clinical Practice (ICH-GCP) or by WHO-GCP standards. Clinical trials require a pre-determined sample size. *Plasmodium falciparum* cases in Vietnam have been steadily decreasing for the last few years highlighting challenges in recruiting a sufficient number of patients into a malaria drug trial. Nevertheless, some respondents suggested it might be possible to organize and conduct trials for TACT in Vietnam.

I think it is possible. Well, if they take the sample size fluctuating between 49 and 61 cases, maybe we can do it…In fact, in Vietnam, given the current situation, we can conduct a clinical trial in areas of Gia Lai [a province of Central Highland in Vietnam], where the number of cases is very high. We can conduct within two years. We can have enough sample size to compare between the current regimen, which is Pyramax [Brand name of PA], and the three-drug regimen [TACT]. (Representative of NGO)

### Requirements for drug registration

Agencies with a Certificate of Eligibility for Pharmacy Business, which permits manufacture, wholesale, export or import of drug/medicinal material, can register drug’s certificates of free sale in Vietnam [20, 21]. The respondents expressed their concerns about the low motivation of agencies to introduce a new anti-malarial given the small market size of Vietnam. They referred to an incentive asymmetry between necessary investments on research, manufacturing, advertising, and regulation while expected profits are limited. This was given as the major reason why domestic manufacturing of anti-malarials in Vietnam has stopped. However, some respondents believed that manufacturers would be interested in manufacturing or investing in TACT for corporate social responsibility rather than for profit.

But in some cases, pharmaceutical companies still do it for the social responsibility to prevent malaria, to eliminate malaria for the community, they could still do it. (Representative of NMP)

Besides such benefits, it will specially lead them to new type of therapy that benefit the patients, the new therapy is more effective than the existing one. (Representative of International Pharmaceutical Company)

For a new drug to be freely distributed, a comprehensive and standardized dossier that includes administrative and technical components, must be submitted to DAV for a certificate. The administrative component refers to application forms, legal documents of the agency and the imported drug. Technical documents are required to have pre-clinical and clinical documents with sufficient data for analysis and justification of influence of Asian racial factors on the safety and efficacy of the drug in order to extrapolate clinical data on the Asian populations. These documents must conform with guidelines given by ICH, the MoH of Vietnam or other organizations recognized by Vietnam [21]. Furthermore, a drug with a new combination of active pharmaceutical ingredients (e.g. TACT) must have sufficient clinical data as required in guidelines of United States Food and Drug Administration (FDA), European Medicines Agency (EMA) [24, 25] or the guideline for clinical development of fixed-dose combination drugs of the WHO. The application package for the certificate often takes several months to prepare.

The Minister of Health can issue a certificate of free sale upon consultancy of the advisory council based on the results of the application assessment. This assessment procedure is coordinated by the DAV. The process can take up to several years to complete, especially when the application needs to be revised and re-submitted multiple times to address questions or concerns on new active ingredients, combination of more than two active ingredients, or indications of serious or frequent side effects. Therefore, well-prepared applications by experienced companies and strict adherence to official guidelines was recommended by respondents when submitting TACT dossiers.

A few additional barriers were identified that previously delayed the approval process for new drugs in Vietnam. The first is the temporary shortage of personnel at the DAV and experts participating in the review councils. Therefore, there are many pending applications on DAV’s waiting list. Second, there are many new registrations every year, and each application add to the waiting list. Third, although the MoH has outlined a roadmap to digitalize the drug registration application, which allows online submission of dossiers, the system has not been implemented yet. The manual management and processing of applications leads to significant increases in workload of the scarce personnel and thereby further increases application timelines.

Another challenge is that the applications are still in paper-based, so they are piled up in the DAV. When I went to work there, I felt overwhelmed with a lot of files around. Therefore, lost files or papers are possibly very high. And I had experiences in lost files twice. And if your files in application are lost, you will have to start over and submit as the beginning. (Representative of International Pharmaceutical Company)

Contrary to legislative documents that indicate a 12-month timespan for a drug’s certificate of free distribution to be issued upon application, it can take up to several years before a drug is approved, as experienced by some respondents.

The manufacturer has started the drug registration process since 2019, the application has been submitted to the DAV, but until now, after 3 years, it has not received approval yet. Meanwhile, Tafenoquine has been one of the drugs in the list of ASEAN's Join-review program to speed up the approval process and is prioritized for approval. (Representative of NGO)

The introduction of a new drug in Vietnam could be shortened if the drugs meet requirements to be granted an import license without certificate of free sale. There are indeed some drugs (e.g. PA for *P. falciparum* treatment or antiretroviral drugs for HIV treatment) that have been licensed for import, to use in Vietnam with a specified quantity without registration for certificate of free distribution, because a) it serves a national health programme, b) it is used as humanitarian aid priority, and/or c) it is to be used for other non-commercial purposes as criteria stated in current Vietnamese pharmaceutical law and the circular of drug registration guideline [20, 21]. If this route is followed, the drug’s dossier would go through an appraisal process of DAV to issue the import license that is said to take about 3 months instead of several years. If TACT goes through the routine drug approval procedures, it will likely not be possible to deploy any triple artemisinin-based combinations on time to meet malaria coordination and elimination ambitions. Therefore, some respondents suggested to apply for an import permit without registration for TACT, as previously done for PA.

### Inclusion in national treatment guidelines

Monitoring and revision of national malaria treatment guidelines is the responsibility of the NMP. Consideration of including a new drug in the treatment guidelines typically is derived from recommendations of the WHO or regional and global policy makers and aims to respond to situations of resistance or reduced effectiveness of current drugs in the country. The NMP will gather experts from the institutes, clinicians from national hospitals, scientists, other stakeholders and WHO representatives in Vietnam to consider options from the list recommended by the WHO, clinical trial data, drug availability and economic efficiency. The draft of new guidelines is developed by the NMP based on consultation of experts at central and local levels and then reviewed by the MoH [26].

The administrative procedures generally take 3 to 4 months for updated guidelines to be approved by the MoH upon submission of final dossiers. However, the timeline for assembling the final dossiers can take up to a year.

Factors that are highly persuasive to malaria policy makers in Vietnam for the introduction of new treatment policies is evidence of resistance to current drugs and convincing evidence of safety and efficacy of the new proposed drug. Additionally, recommendation by the WHO, achieving WHO prequalification status, approval by Australia Therapeutic Goods Administration (TGA) or US FDA are considered important enablers. Policymakers would consider drugs that are licensed by the Ministry of Health, commercially available, low-cost, and accepted by donor funders. Respondents stated that facilitating the communication between policymakers, pharmaceutical companies and donors is important to promote the introduction of a drug. Activities carried out by NGOs for promotion of tafenoquine were highlighted.

Firstly, providing information to experts in Vietnam, such as the results of scientific research, the results of clinical trials. Secondly, providing the experts here or the decision-makers information to let them know about, for example, how the process of Tafenoquine approval in other countries was like. Then providing information about the process that is called PQ approval, such as process of WHO Prequalification approval. As we have involved in some Global projects, for example, they have got some connections with the World Health Organization so that we could understand the processes of submission and approval for introducing the guidelines of the World Health Organization… organized big seminars to provide information for both sides. For policy makers and health workers, they paid attention to the cost, the approval of drug use in Vietnam. To the manufacturer, our project also conducted seminars for them so that the manufacturer could realize the need of the drug in Vietnam. (Representative of NGO)

Almost all respondents indicated that it would not be challenging for TACT to be included in national guidelines if its safety and efficacy can be demonstrated through clinical data collected in Vietnam, and if it is included in the WHO anti-malarial treatment guidelines.

### Roll-out of a change in the treatment policy

Based on their experiences from the last changes in malaria treatment, respondents suggested a systematic training of trainers on new treatment therapy be conducted from the central to community level because malaria treatment mainly takes place at community health stations. Comprehensive training must be organized for hospitals and preventive facilities in order to have a sound understanding of new treatments including adverse events and how to handle them. The NMP usually prioritizes the training in provinces with high malaria prevalence first, and then in provinces with risk of imported malaria cases. The training should be available digitally through public guidelines or as video content.

I don't think it challenges. It only requires human resources from three Institutes [NIMPE, IMPE in Ho Chi Minh and Quy Nhon cities], who will conduct trainings for the provinces, then the provinces themselves will train down to the lower levels like that (Representative of NMP)

The respondents believed that new therapies, such as TACT, would be easily accepted by prescribers and patients if it is convenient to use as a single dose per day for a short period, available as a fixed dose combination, and with no adverse effects. Surveillance of adverse effects should be based on national drug information and adverse drug reaction system [27] and NMP’s surveillance systems as regulations.

### The current need for new malaria treatment therapies

As indicated in interviews, DHA-PPQ used to be prescribed for uncomplicated *P. falciparum* malaria in provinces where evidence of drug resistance had not been detected. However, in 4 provinces with high rate of resistance, DHA-PPQ had been completely replaced by PA from 2020. PA is now the only treatment available in Vietnam because of external funds and scarcity of domestic DHA-PPQ. Although treatment guidelines have not yet been revised, many national programme leaders consider PA to be the first-line treatment for malaria because it has high efficacy [1, 11]. The NMP leaders plan to change this guideline in the near future.

After the combination of DHA-PPQ, the National Malaria Program and the World Health Organization [Representative in Vietnam] had meetings and decided to change the antimalarial from DHA-PPQ to use a combination called Pyramax [PA], as you probably know, Pyramax has been used for 2 recent years… In the near future, it is likely that new documents, new regulations will be issued. (Representative of NMP)

Respondents confirmed their awareness of the emergence and spread of artemisinin resistance in Vietnam, but the situation was considered somewhat more positive than in other countries in the region. The treatment failure rate with DHA-PPQ reached up to 60%, which was also confirmed by recent therapeutic efficacy studies [28, 29]. Artesunate–mefloquine is currently not widely used in the NMP. According to the Annual Report of NMP for 2022, the day 3 positivity was 40% after treatment with artesunate–mefloquine [1].

Many respondents were confident about the efficacy of the newly introduced treatment, PA, and expected to rely on current artemisinin-based combinations until *P. falciparum* malaria elimination is achieved. They perceived that introduction of new treatment at this stage is not an urgent necessity. They also referred to the fact that there have not been any new treatment policies for *P. falciparum* recommended by the WHO other than the current artemisinin-based combinations.

PA is procured internationally by The Global Fund to Fight AIDS, Tuberculosis and Malaria and provided to the countries in the region. Although the cost for procurement of drug and logistics would not be a burden, the self-procurement of a small quantity of drugs for diminishing treatment needs would be challenging for these countries to attract suppliers, as indicated by respondents. Therefore, in the future, if bordering countries of Cambodia and Lao PDR change their treatment policies due to the spread of drug resistance in these countries, Vietnam would need to consider accordingly.

### Timeline to introduce TACT and malaria elimination target

Although Vietnam is making good progress towards the target of eliminating *P. falciparum* by 2025, some respondents were not optimistic that Vietnam can accomplish this target within the time frame because these respondents believed that the last cases would be the hardest to eliminate.

To introduce any drug into a market like that will be, will be quite difficult, very time – consuming and it may take a few years so it might be that the introduction process is not finished but the program is already, already [laughter] over. (Representative of NGO)

it seems impossible to do that [malaria elimination] so in a few, a few years, by around 2025–2026 (Representative of NGO)

The respondents were concerned that resistance to the newly introduced PA may emerge in the next few years even though it is currently effective. They cited concerns related to the movement of mobile migrant population among countries in the Greater Mekong Subregion leading to the spread of drug resistance, and the drug pressure on PA when it becomes the only treatment choice in the countries. Therefore, respondents prefer access to alternative treatments as a backup if PA starts to fail or if there is a resurgence after elimination is achieved.They indicated that to their knowledge there are no new drug compounds expected on the market within the next years, and therefore TACT would be useful because they it could be deployed rapidly. Respondents agreed that moving towards introducing TACT would require timely preparation of procedures.

But we only give one kind of drug (PA) for those 200 cases, then, the pressure of the drug on the population of parasites that already has multi-resistance. Therefore, the likelihood of resistance now is much higher than in the past (Representative of NGO)

So, if the efficacy of the current drug decreases faster than they expected, they must have another one to cover. So, I think now is the time to introduce it, not so that it can be used immediately, but for the time we need to prepare the necessary procedures and works before it can officially be used. (Representative of NGO)

Other respondents indicated that TACT should be used in the country as soon as possible to support the *P. falciparum* elimination target.

TACT is a, a very sharp knife for the current situation of drug resistance in Vietnam and also in the region. It’s an alternative that’s worth paying attention to and following and should be put in use as soon as possible so we can, can manage immediately any case detected. That is, to eliminate it, to not let it reoccur and spread out, otherwise it would be more difficult to control. So, I think that in term of treatment, case-by-case treatment is very necessary these days. (Representative of NGO)

Indeed, to allow for TACT’s full potential in preventing drug resistance, TACT would ideally be deployed even in settings where ACT remains efficacious [13, 18]. This is demonstrated through recent mathematical modelling studies predicting that switching to TACT could substantially delay the emergence and evolution of artemisinin-resistant alleles and reduce treatment failures [18].

## Limitations of the study

Selection of respondents occurred in close collaboration with NIMPE. Nevertheless, some other important stakeholders (such as representatives from DAV, WHO and The Global Fund in Vietnam) with different perspectives did not join the interviews, despite the efforts of research team to reach out to them. Interviews were conducted in Vietnamese and translated into English. Though the translation was done by professional translators with experience in the medical domain, the translation bias could have occurred.

## Conclusions and way forward

TACT is considered as a useful backup in case current artemisinin-based combinations would fail, and as a strategy to prevent the re-establishment of malaria. Exploring the processes related to changing malaria treatment policy in Vietnam raised three challenges that need strategic consideration before introducing TACT.

First, malaria policy makers in Vietnam expressed their reliance on current ACT in reaching malaria elimination targets. However, they agreed that TACT could be considered as alternative treatment in case PA starts to fail. The respondents indicated that evidence of TACT’s safety, tolerability and efficacy should actively be disseminated to national decision makers, regulators, and suppliers. This requires early stakeholder engagements through seminars, reports, and additional advocacy activities. These efforts could convince policy makers of the potential benefits of deploying TACT even though current ACT remains effective, which could in turn prevent similar delays in introducing TACT, as was previously observed with ACT implementation.

Second, national legislative documents suggest that any drug that qualified safety and efficacy assessments from clinical trials can be considered for circulation in Vietnam, regardless of where the clinical trials were conducted. Nevertheless, this was not known by national policy makers who were convinced that evidence of efficacy and safety of TACT in Vietnamese populations would be required. While exploring options to conduct clinical trials in Vietnam (amidst declining prevalence of malaria) is important, measures to disseminate the information in national legislative documents must be implemented. WHO prequalification and inclusion in WHO treatment guidelines were cited as important factors, to give governments more confidence about their decision and, therefore, TACT manufacturers must seek these endorsements to ensure timely availability.

Third, the routine procedure of registration would be lengthy. Application procedures for an import permit of TACT without registration was suggested as a strategy to reduce introduction timelines. This strategy was previously applied for PA because a new therapy was urgently needed after DHA-PPQ failures occurred. Adopting a similar strategy for TACTs could ensure timely registration and deployment of triple artemisinin-based combinations to support reaching malaria elimination goals and/or to prevent a resurgence of falciparum malaria [18].

### Supplementary Information

**Additional file 1.** Qualitative Interview Guide for Stakeholders in Vietnam.

## Data Availability

The datasets used and/or analysed during the current study are available from the corresponding author on reasonable request.

## Abbreviations

ACT: Artemisinin-based Combination Therapy
ASMQ: Artesunate–mefloquine
DAV: The Drug Administration of Vietnam
DeTACT: Development of Triple Artemisinin Combination Therapies project
DHA-PPQ: Dihydroartemisinin–piperaquine
EMA: European Medicines Agency
GCP: Good Clinical Practices
GFATM: The Global Fund to fight AIDS, Tuberculosis and Malaria
ICH: International Council for Harmonization of Technical Requirements for Pharmaceuticals for Human Use
IMPEs: Institutes of Malariology, Parasitology and Entomology
NGO: Non-Governmental Organization
NIMPE: National Institute of Malariology, Parasitology and Entomology
NMP: National Malaria Programme
MoH: Ministry of Health
PA: Artesunate-pyronaridine
TACT: Triple artemisinin-based combination therapy
TGA: Australia Therapeutic Goods Administration
US FDA: United States Food and Drug Administration
WHO: World Health Organization
