# Supplementary material for: Exploring the feasibility of introducing triple artemisinin-based combination therapy in the malaria treatment policy in Vietnam
Source: Malar J. 2023 Oct 28;22:326. doi: 10.1186/s12936-023-04763-4 (PMC10613363; doi:10.1186/s12936-023-04763-4)
Supplement: Supplementary file 1 — Additional file 1. Qualitative Interview Guide for Stakeholders in Vietnam. [file 12936_2023_4763_MOESM1_ESM.docx]

# Additional file 1: Annex S1: Interview guide – Vietnam

Background of participant

- Could you briefly introduce yourself: Please tell a bit about your organization and your position?

1. General questions

- *Ask suitable question based on background respondent*
- What can you tell about the current situation of artemisinin and partner drug resistant malaria in Vietnam?
- What are your thoughts on introducing TACTs in Vietnam?

1. Implementation barriers

- *In a recent delphi study we have asked malaria experts to identify implementation barriers for introducing TACTs in Southeast Asia. We will present these implementation barriers to you and ask your opinion about them. Our goal is to explore the causes behind each barrier and to brainstorm about potential strategies to overcome them:*

*The table below presents the implementation barriers for introducing TACTs in Southeast Asia. Please ask respondent:*

- - For each implementation barrier [X] ask:
    - What is the current situation?
    - What is the implication for TACTs?
  - Then ask follow-up questions

| **X** | **Implementation barriers** | **Explanation of the implementation barrier** |
| --- | --- | --- |
|  | WHO and global policy support | Introducing TACTs will require support from the WHO and other global decision makers.   - *What is the role of the WHO in malaria policy in Vietnam?* - *What would be challenges of WHO support for introducing TACTs in Vietnam?* - *What would be challenges and what are solutions?* |
|  | Donor funder support | Introducing TACTs will require support by donor funders to cover implementation costs and potential price increases.   - *What is the role of donor support in malaria in Vietnam?* - *What would be the challenges of donor fund support for introducing TACTs in Vietnam?* - *What would be solutions to these challenges?* |
|  | National policy support | Introducing TACTs will require support from national malaria control programs and other national decision makers.   - *What are national policies around malaria drug resistance in Vietnam?* - *What would be the challenges to change national policy for introducing TACTs in Vietnam?* - *What would be solutions to these challenges?* |
|  | Availability of fixed-dose combination (FDC) TACTs | Ensuring timely development and production of fixed-dose combination (FDC) for TACTs is a challenge.   - *How are malaria medicines prescribed now? How important is it to have fixed-dose combinations (pills merged)?* - *What are the challenges for having fixed-dose combinations for TACTs* - *What would be solutions to these challenges?* |
|  | Collecting safety and efficacy data | Introducing TACTs will require efficacy and safety data. It can be difficult to collect such data.   - *What kind of safety/efficacy data is required for medicines in Vietnam?* - *What are challenges for safety/efficacy data of TACTs in Vietnam? What procedures need to be followed?* - *What would be solutions to these challenges?* |
|  | Regulatory approval | Obtaining timely regulatory approval for introducing TACTs in Vietnam.   - *Can you explain what the process of drug regulation looks like? What are the normal procedures for registration?* - *What would be challenges for registration/ regulatory approval for TACTs?* - *What would be solutions to these challenges?* - *How long would the regulatory procedure for TACTs normally take?* |
|  | Supply chain logistics | Introducing TACTs will require changes in import, procurement and supply routes for the introduction of TACTs.   - *What do supply chains for malaria medicines in Vietnam look like?* - *What are the challenges for integrating TACTs in the supply chains?* - *What would be solutions to these challenges?* |
|  | Private sector (drug store etc) engagement | Introducing TACTs will require engaging the (informal) private sector in TACTs deployment and creating demand beyond official programs.   - *What is the role of the private sector in malaria treatment in Vietnam now?* - *What would be challenges in engaging private sector in TACTs in Vietnam?* - *What would be solutions to these challenges?* |
|  | Stockpile management | Introducing TACTs will require managing stocks of ACT and contract deals with ACT producers.   - *What are current practices for stockpile management of malaria medicines in Vietnam?* - *What would be challenges for stockpile management of TACTs in Vietnam?* - *What would be solutions to these challenges?* |
|  | Intensified prescriber (doctor, pharmacist training) | Introducing TACTs will require intensified training requirements for correct prescription   - *How are prescribers being trained for malaria now?* - *What training would be required if we change to TACTs?* - *What would be solutions to these challenges?* |
|  | Community acceptance (patients) | Introducing TACTs may require clear communication messages and tackling potential misconceptions about TACTs.   - *What are current challenges with community acceptance of malaria medicines?* - *What challenges do you expect for community acceptance of TACTs?* - *What would be solutions to these challenges?* |
|  | Set up surveillance systems | Introducing TACTs will require the setup of surveillance systems to monitor drug resistance and adherence to TACTs.   - *What malaria surveillance systems do exist now in Vietnam?* - *What types of surveillance systems would be required for TACTs? What challenges would you expect to setup these systems?* - *What would be solutions to these challenges?* |
|  | Set up pharmacovigilance systems | Introducing TACTs will require the setup of a pharmacovigilance system.   - *What malaria pharmacovigilance systems do exist now in Vietnam?* - *What pharmacovigilance systems would be required for TACTs? What challenges would you expect to setup these systems?* - *What would be solutions to these challenges?* |

1. Other questions

- Did you miss any implementation barriers for introducing TACTs on this list?
- What would be appropriate prices for TACTs in Vietnam?
- How would introducing TACTs be different than rotating with current ACTs?
- What effect would the introduction of TACTs have on malaria elimination strategies?
- Do you have suggestions for other people we need to interview?

Thank you for participating in this interview.

End of interview
